# Percutaneous cholecystostomy as a definitive treatment for moderate and severe acute acalculous cholecystitis: a retrospective observational study

**DOI:** 10.1186/s12893-021-01411-z

**Published:** 2021-12-27

**Authors:** Bai-Qing Chen, Guo-Dong Chen, Feng Xie, Xue Li, Xue Mao, Bao Jia

**Affiliations:** 1 Department of Nuclear Medicine, The People’s Hospital of Liaoning Province, 33 Wenyi Road, Shenhe District, Shenyang, 110016 China; 2 Department of Radiology, Panjin Liaohe Oilfield Gem Flower Hospital, 26 Yingbin Road, Xinglongtai District, Panjin, 124010 China

**Keywords:** Percutaneous cholecystostomy, Acute acalculous cholecystitis, Coronary disease, Charlson Comorbidity Index

## Abstract

**Background:**

In this study, we aimed to investigate risk factors for the relapse of moderate and severe acute acalculous cholecystitis (AAC) patients after initial percutaneous cholecystostomy (PC) and to identify the predictors of patient outcomes when choosing PC as a definitive treatment for AAC.

**Materials and methods:**

The study population comprised 44 patients (median age 76 years; range 31–94 years) with moderate or severe AAC who underwent PC without subsequent cholecystectomy. According to the results of follow-up (followed for a median period of 17 months), the data of patients with recurrence versus no recurrence were compared. Patients were divided into the death and non-death groups based on patient status within 60 days after PC.

**Results:**

Twenty-one (47.7%) had no recurrence of cholecystitis during the follow-up period after catheter removal (61–1348 days), six (13.6%) experienced recurrence of cholecystitis after PC, and 17 (38.6%) patients died during the indwelling tube period (5–60 days). The multivariate analysis showed that coronary heart disease (CHD) or congestive heart failure (odds ratio [OR] 26.50; 95% confidence interval [CI] 1.21–582.06; *P* = 0.038) was positively correlated with recurrence. The age-adjusted Charlson comorbidity index (OR 1.53; 95% CI 1.08–2.17; *P* = 0.018) was independently associated with 60-day mortality after PC.

**Conclusions:**

Our results suggest that CHD or congestive heart failure was an independent risk factor for relapse in moderate and severe AAC patients after initial PC. AAC patients with more comorbidities had worse outcomes.

## Introduction

Acute acalculous cholecystitis (AAC) is a kind of acute cholecystitis without stone formation in the gallbladder. The etiology is multifactorial and may be caused by bile stasis or ischemia (or both). Bile stasis can result from fasting, ileus or total parenteral nutrition (TPN) [1]. The clinical features of pediatric AAC present significant differences compared to adult AAC [2]. Therefore, we only discuss adult AAC in this study.

Currently, percutaneous cholecystostomy (PC) is used more often for moderate and severe AC patients who are not considered suitable for early surgery. According to recently reported results, cholecystectomy appears to be necessary in these patients even after PC. The CHOLOLATE trial demonstrated that laparoscopic cholecystectomy (LC) compared with PC reduced the rate of major complications in high-risk patients with acute cholecystitis, including a lower incidence of recurrent biliary disease [3]. The Tokyo Guidelines 2018 (TG18) also recommends that “if a patient is deemed capable of withstanding surgery for AC, we propose early surgery regardless of exactly how much time has passed since onset” [4]. However, the subject of CHOLOLATE was acute calculous cholecystitis (ACC), and the TG18 does not separate ACC from AAC. ACC patients have stones, which are a risk factor for AC recurrence, while AAC patients do not have this concern [5]. Some AAC patients may present with dysfunctional gallbladder syndrome, biliary dyskinesia due to prolonged NPO status, viral infections, malignancies, and other metabolic derangements. If PC is performed in a timely manner, then the physiological structure of the gallbladder will not be significantly damaged, and the function of the gallbladder can recover after PC [6].

To date, several studies have observed and reported the outcomes of AAC patients who did not undergo cholecystectomy after percutaneous cholecystostomy treatment [6–9]. Noh et al. evaluated the clinical outcomes of PC in 271 patients with AAC, which is the largest cohort size ever reported. They believed that PC alone can be a definitive treatment option for the majority of AAC patients, but even so, two of the 88 patients in the catheter removal group experienced cholecystitis recurrence. Other researchers have reported similar findings—that a few AAC patients had recurrence after PC alone [10, 11]—so it is necessary to investigate the risk factors for recurrence. The TG18 recommends LC, and conservative treatment should be performed first in mild AAC patients [4], so these patients were excluded from our study.

The goal of this study was therefore to investigate risk factors for the relapse of moderate and severe AAC patients after initial PC and to identify the predictors of patient outcomes when choosing PC as a definitive treatment for AAC.

## Materials and methods

### Patient population

Detailed information regarding patients was retrieved from the People’s Hospital of Liaoning Province. All procedures performed in studies involving human participants were in accordance with the ethical standards of the institutional and national research committee and with the 1964 Declaration of Helsinki and its later amendments or comparable ethical standards. This retrospective study was approved by the Ethics Committee of the People’s Hospital of Liaoning Province and strictly adhered to the tenets of the Declaration of Helsinki (code of Ethical approval for scientific research project: 2020 Ethical Scientific Research Approval No. HS015). Given the retrospective design, a waiver of participant consent was granted by the ethics committee.

The electronic medical database of our institution was searched for records of 241 acute cholecystitis patients who underwent PC for the first time between January 1, 2017, and February 29, 2020. The diagnosis of AAC was based on clinical symptoms and signs (fever, abdominal pain, a positive sonographic Murphy’s sign, or elevated inflammatory markers such as white blood cells) and radiologic studies of abdominal US (ultrasonography), MRCP (magnetic resonance cholangiopancreatography), CT (computed tomography). On imaging, the absence of gallstones in combination with the enlargement and thickening of the gallbladder wall, a positive sonographic Murphy’s sign, and pericholecystic fluid were regarded as signs of AAC. The diagnostic criteria and severity grading of acute cholecystitis were based on the TG18 [12]. The patients were divided into Grade I (mild) AC, Grade II (moderate) AC, and Grade III (severe) AC. Patients with concurrent or secondary pancreatitis (*n* = 35) and pancreatic trauma (*n* = 1) were excluded. Patients who had gallstones (including sludge) identified by imaging or surgical specimens (*n* = 135) were also excluded. Among the remaining 70 patients with AAC, those with mild AAC (*n* = 10) and with concurrent common bile duct stones (*n* = 6) were also excluded. According to the follow-up results, patients who were lost to follow-up (*n* = 6) and those who underwent cholecystectomy after the resolution of AAC during follow-up (*n* = 4) were also excluded. The four patients all had clinical remission after PC and no recurrence before cholecystectomy. A total of 44 patients (mean age, 76 years; range 31–94 years; male, n = 28) ultimately comprised the study population.

### Percutaneous cholecystostomy

Our policy when faced with moderate or severe AAC is to monitor of respiration and hemodynamics, as well as sufficient intravenous fluid and electrolyte infusion and electrolyte correction and treatment with broad-spectrum antimicrobials. Patients whose condition worsens or fails to improve clinically within 48 h are reevaluated by surgeons, anesthesiologists and interventional radiologists, and a decision is made depending on the patient’s preference for either emergent LC or PC. PC was performed by an experienced interventional radiologist who had 15 years of experience. In brief, using local anesthesia and sterile techniques, a needle was introduced via the transhepatic (*n* = 36) or transperitoneal (*n* = 8) route under ultrasonographic guidance into the gallbladder. A 0.035-inch guidewire was inserted, followed by placement of an 8-Fr pigtail catheter. The transhepatic route is preferred in our institution because it possesses greater catheter stability, a lower rate of bile leakage and quicker maturation of the tract [7]. A mark was made on the drainage tube so that tube prolapse could be detected promptly, and the drainage bag was replaced twice a week. We do not routinely flush the tube in AAC patients. When bile drainage decreases significantly, the catheter is flushed with a syringe filled with normal saline. Patients received intravenous antibiotics before and after PC, and the duration of intravenous antibiotics was determined by the attending doctor. In our institution, patients were discharged from the hospital after meeting the standard of clinical effectiveness (If the patient only has cholecystitis, usually three days after PC) and were readmitted after four weeks to remove the tube. The indication for removal of the PC catheter was as follows: laboratory values returned to normal, ultrasonography or CT imaging indicated improvement, and the PC tube was clamped for 2–3 days without recurrent symptoms of AAC.

### Measured/defined variables

We recorded patient demographic data, the last laboratory examination before PC, maximum body temperature within 3 days before PC, laboratory examination 3–5 days after PC (the highest value was selected when there were multiple review results), maximum body temperature 3–5 days after PC, changes in preoperative and postoperative complaints of abdominal pain, type and length of antibacterial treatment before PC, postoperative complications, length of stay, ICU duration and drainage duration. The classification of comorbidities was defined by Charlson comorbidity index (CCI). After PC, patients were followed up until AAC-related readmission, death, or loss to follow-up occurred.

We obtained age-adjusted Charlson Comorbidity Index (aCCI) scores and clinical efficiency from the data we collected [13]. In our institution, temperature and the changes in abdominal pain were collected daily, and laboratory values were reexamined 3–5 days after PC. Combined with relevant definitions of other studies [7, 14–16] and the situation of our institution, clinical effectiveness was defined as the improvement in at least two of the following three items: abdominal pain, white blood cell (WBC) count, and temperature within 5 days after PC and no recurrence within at least 30 days. The definition of improvement of abdominal pain was that the patient felt relief from abdominal pain after PC. Improvement in WBC count is defined as a reduction in postoperative count compared to the preoperative. Improvement in temperature was defined as normal or lower than preoperative after PC. AAC recurrence was diagnosed based on clinical signs (fever and right upper quadrant abdominal pain) and abdominal ultrasonography (US)/computed tomography (CT) findings.

### Statistical analysis

Categorical data are expressed as counts and proportions, and continuous data are expressed as the median and quartile range. Comparisons between groups were carried out using the Wilcoxon rank-sum test for continuous and ordinal variables and the Chi^2^ test or Fisher’s exact test for categorical variables. To assess risk factors for relapse and predictive factors for mortality within 60 days after PC, logistic regression analyses were used to calculate the odds ratio (OR) with 95% confidence intervals (CI). Variables with a *P*-value ≤ 0.05 in the univariate analysis were selected for the multivariate analysis. All statistical analyses were performed using SPSS software (version 24.0; Armonk, NY).

## Results

### Clinical features

A total of 44 patients (28 male) with a median age of 76 years were identified. Eighteen patients had moderate AAC (40.9%). The median CCI was 2 (range 0–9). Thirty-one patients with a primary diagnosis of AC at admission and 13 patients who developed AAC during hospitalization were included. Of the 13 patients, five had AAC after fasting, eating difficulties or TPN, three had AAC after intestinal obstruction, two had AAC after major surgery, one had AAC after traffic accident, one had AAC after biliary obstruction, and one had AAC after chronic renal failure (CRF). The average length of stay of 44 patients was 22 days, and the mean post-operative length of hospital stay was 15 days. Sixteen patients were transferred from the general ward to the intensive care unit (ICU) and had a median time in the ICU of 5 days (range 1–33 days). Eleven cases were transferred to the ICU before PC, 10 of which were severe AAC. Five cases were transferred to ICU after PC, three of which were severe AAC. Nine patients had jaundice before PC. The causes of jaundice were biliary obstruction caused by malignant tumors in three cases, splenic rupture due to traffic accidents in one case, and hepatitis C in one case. The remaining four cases did not have any reasons for jaundice other than AAC, and their serum total bilirubin levels decreased significantly within 5 days after PC. Nine patients had coronary heart disease (CHD), of whom five had prior myocardial infarction. Bile was collected from 24 (54.5%) patients and cultured, and nine (37.5%) patients had positive cultures. The bile culture results included gram-negative rods (*n* = 5) and gram-positive cocci (*n* = 4). Twenty-four patients were administered only a single antimicrobial preoperatively. Seventeen patients were administered multiple antimicrobials containing at least two of the following: cephalosporin, carbapenem, fluoroquinolone, vancomycin, and aminoglycosides. Two patients did not receive intravenous antibacterial treatment before PC. One patient was treated at another hospital before undergoing PC, and the medication administered at the other facility is unknown. Of note, a total of 21 patients were treated with carbapenem before PC. For patients with only AC, or with positive bile culture results, the de-escalation of antimicrobial therapy will be taken after PC. Three patients received intravenous medication for pain caused by AAC before PC. Another three patients received intravenous pain medication for pain caused by AAC after PC.

The clinical characteristics and demographics of the patients are described in Table 1.

**Table 1:** Demographic and clinical characteristics of the patients

Variable	AAC patients receiving PC treatment (n = 44)
Sex [male (%)]	28 (63.6%)
Age (years)	76 (65–82)
Length of stay (days)	15 (8–27)
Intensive care unit stay	16 (36.4%)
Grade II/III	18 (40.9%)/26 (59.1%)
Cardiovascular dysfunction	8 (18.2%)
Neurological dysfunction	14 (31.8%)
Respiratory dysfunction	10 (22.7%)
Renal dysfunction	16 (36.4%)
aCCI	5 (4–6)
Charlson comorbidity	
Prior myocardial infarction	5 (11.4%)
Congestive heart failure	4 (9.1%)
Diabetes	12 (27.3%)
Cerebrovascular disease	11 (25.0%)
Nonmetastatic solid tumor	7 (15.9%)
Metastatic solid tumor	7 (15.9%)
Moderate or severe renal disease	4 (9.1%)
Ulcer disease	3 (6.8%)
Preoperative jaundice	9 (20.5%)
Common bile duct obstruction	3 (6.8%)
Body temperature (℃)	38.1 (37.1–39.0)
Preoperative fever (≥ 37.7℃)	27 (61.4%)
Initial laboratory values	
Platelets (× 10^9^ L)	173.5 (117.5–322.0)
WBC counts (× 10^9^ L)	10.3 (7. 5–16.0)
Neutrophil granulocytes (%)	83.2 (76.2–92.6)
ALT (U/L)	30.5 (19.3–62.0)
STB (μmol/L)	19.5 (13.0–30.7)
CB (μmol/L)	1.6 (0–10.5)
UCB (μmol/L)	6.6 (3.3–12.6)
Antibacterial treatment before PC	
Cephalosporin only	14 (31.8%)
Carbapenem only	9 (20.5%)
Fluoroquinolone only	1 (2.3%)
Multi-drug combination	17 (38.6%)

*aCCI* age-adjusted Charlson comorbidity index; *WBC* White blood cells; *ALT* alanine aminotransferase; *STB* serum total bilirubin; *CB* conjugated bilirubin; *UCB* unconjugated bilirubin

### Follow-up results

We obtained the follow-up outcomes of 44 patients after PC (range 5–1378 days). Twenty-one (48%) patients had no recurrence of cholecystitis during the follow-up period after PC (range 114–1378 days) and after tube removal (range 61–1348 days). Six (13.6%) patients experienced cholecystitis recurrence after PC. Two (33.3%) patients had recurrence during indwelling drainage tubes, of whom one had recurrence 11 days after PC, and the other changed the catheter six months after PC and had recurrence 46 days after catheter change. Symptoms were relieved after antibiotics alone. Four (66.7%) patients had recurrence after tube removal, of whom one experienced recurrence in the ninth month after tube removal, and the others experienced recurrence in the fifth month. Clinical resolution of AAC was achieved after antibiotics alone, except in one patient who died in the hospital. The cause of death was uncontrolled septic shock. The date of tube removal was clearly documented for 40 patients, with a median drainage duration of 29 days (range 5–252 days).

PC was technically successful in all patients. The clinical effectiveness rate was 72.7% (32/44) at 5 days after surgery. Only two patients (2/44, 4.5%) with PC-related complications had catheter dislocations.

We compared the preoperative clinical features and postoperative curative effects between the group that had no recurrence of AAC during the follow-up period (nonrecurrence group; *n* = 21) and the group that experienced recurrence of AAC (recurrence group; *n* = 6). The results of the univariate and multivariate analyses of the predictive factors for recurrence are shown in Table 2. The multivariate analysis showed that CHD or congestive heart failure (CHF) on admission was positively associated with recurrence (OR 26.50; 95% CI 1.21–582.06; *P* = 0.038). WBC counts and neutrophil granulocytes were not found to be associated with recurrence in the multivariate analysis.

**Table 2:** Comparison of the nonrecurrent and recurrent patient groups

Variable	No recurrence (n = 21)	Recurrence (n = 6)	Univariate analysis *P*-value	Multivariate analysis OR(95% CI) *P*-value
Sex [male (%)]	15 (71.4%)	2 (33.3%)	0.15	
Age (years)	76 (63–83)	79 (66–83)	0.52	
Length of stay	15 (10–27)	14 (9–36)	0.82	
TG18 grade				
II	9 (42.9%)	3 (50.0%)	1	
III	12 (57.1%)	3 (50.0%)		
aCCI	4 (3–5)	5 (4–5)	0.13	
Preoperative body temperature (℃)	38.0 (37.2–39.2)	37.5 (36.6–38.5)	0.15	
Initial laboratory values				
Platelets (× 10^9^ L)	172.0 (114.0–294.0)	163.50 (101.75–284.25)	0.77	
White blood cells (× 10^9^ L)	11.4 (8.6–17.2)	6.2 (4.2–9.7)	0.031	
Neutrophil granulocytes (%)	86.1 (75.5–93.0)	74.6 (68.0–82.3)	0.047	
ALT (U/L)	39.0 (24.5–92.5)	27.0 (21.7–91.8)	0.56	
STB (μmol/L)	20.0 (13.6–33.9)	17.0 (7.9–23.8)	0.24	
CB (μmol/L)	1.4 (0–10.2)	1.9 (0–5.4)	0.62	
UCB (μmol/L)	9.4 (3.7–13.8)	6.2 (3.5–18.5)	0.86	
CHD or CHF	3 (14.3%)	4 (66.7%)	0.024	26.50 (1.21–582.06) 0.038
Diabetes	3 (14.3%)	3 (50.0%)	0.10	
Cerebrovascular disease	4 (19.0%)	3 (50%)	0.29	
Malignancy	4 (19.0%)	2 (33.3%)	0.59	
Clinical effectiveness	16 (76.2%)	5 (83.3%)	1	

*aCCI* age-adjusted Charlson comorbidity index; *WBC* White blood cells; *ALT* alanine aminotransferase; *STB* serum total bilirubin; *CB* conjugated bilirubin; *UCB* unconjugated bilirubin; *CHD* coronary heart disease; *CHF* congestive heart failure

Approximately one-third of the patients died during the tube indwelling period. The shortest time from PC to death was 5 days, and the longest time was less than 60 days. To explore the predictive factors for the 60-day mortality rate after PC, we compared the preoperative clinical features and postoperative curative effects between the group of patients who survived more than 60 days after PC (non-death group; *n* = 27) and the group of patients who died within 60 days after PC (death group; *n* = 17). The causes of death were terminal malignant tumors in eight cases, sepsis or disseminated intravascular coagulation as a result of AAC in two cases, pneumonia in two cases, diabetic ketoacidosis in one case, and multiple organ failure as a result of chronic kidney disease in two cases. The specific causes of death in two cases were not clear. No causes of death were dependent upon the PC.

The preoperative clinical features and postoperative curative effects were compared between the non-death group (*n* = 27) and the death group (*n* = 17) (Table 3). When compared to the death group, we found a highly significant difference for aCCI (*P* = 0.0010) and preoperative ALT (*P* = 0.014). There was no significant difference in clinical efficiency between the non-death group and the death group (77.8% vs. 64.7%, *P* = 0.55). In multivariate analysis, aCCI (OR 1.53; 95% CI 1.08–2.17; *P* = 0.018) was positively correlated with mortality. ALT itself was not found to be associated with mortality in the multivariate analysis.

**Table 3:** Comparison of the nondeath and death groups

Features	Nondeath (n = 27)	Death (n = 17)	Univariate analysis *P*-value	Multivariate analysis OR(95% CI) *P*-value
Sex [Male (%)]	17 (62.9%)	11 (64.7%)	0.91	
Age (years)	76 (64–82)	74 (70–80)	0.84	
Length of stay	15 (10–26)	14 (8–32)	0.98	
TG18 grade				
II	12 (44.4%)	6 (35.3%)	0.55	
III	15 (55.6%)	11 (64.7%)		
aCCI	4 (3–5)	7 (5–9)	0.0010	1.53 (1.08–2.17) 0.018
Preoperative body temperature (℃)	38.0 (36.9–39.0)	38.3 (37.3–39.3)	0.41	
Initial laboratory values				
Platelets (× 10^9^ L)	172.0 (111.0–290.0)	249.0 (124.5–374.5)	0.21	
White blood cells (× 10^9^ L)	9.32 (6.41–16.04)	10.66 (7.65–17.08)	0.56	
Neutrophil granulocyte (%)	82.4 (74.3–92.6)	83.4 (81.0–93.0)	0.32	
ALT (U/L)	38.0 (23.3–92.0)	22.0 (13.3–34.0)	0.014	
STB (μmol/L)	20.0 (13.4–31.1)	17.3 (12.1–32.9)	0.96	
CB (μmol/L)	1.4 (0–8.0)	2.9 (0–13.3)	0.69	
UCB (μmol/L)	7.8 (3.9–15.5)	6.3 (2.4–12.5)	0.56	
Clinical effectiveness	21 (77.8%)	11 (64.7%)	0.55	

*aCCI* age-adjusted Charlson comorbidity index; *WBC* White blood cells; *ALT* alanine aminotransferase; *STB* serum total bilirubin; *CB* conjugated bilirubin; *UCB* unconjugated bilirubin

## Discussion

To date, there are few reports regarding risk factors for AAC relapse, but several risk factors for AAC have been identified [1, 17]. We speculated that risk factors for AAC that were not eliminated after the remission of AAC symptoms may be risk factors for AAC relapse. Therefore, CHD or CHF, underlying malignancy, diabetes and other incurable comorbidities were compared between the nonrecurrent and recurrent patient groups. Our results showed that the presence of CHD or CHF at the time of PC placement was independently associated with recurrence. CHD and CHF are cardiovascular diseases that can cause hypoperfusion and ischemia of the gallbladder-related blood supply and are incurable at present. If patients who undergo PC have CHD or CHF, they may need more active management to reduce the rate of recurrence, such as long-term indwelling drainage tubes or cholecystectomy (if possible). Park et al. reported that underlying malignancy was positively correlated with AC recurrence [5], but our results indicated that there was no significant difference in underlying cancer between the nonrecurrent and recurrent patient groups. In addition, there may be predictors of recurrence in the form of clinical features that we did not assess. Bhatt et al. reported that purulence in the gallbladder and calculous cholecystitis at the time of PC placement were related to a higher probability of recurrence of AC in a univariate analysis [18]. A total of three patients with suppurative cholecystitis were included in our study, and they did not experience recurrence during the follow-up period.

None of the deaths in the death group in this study were related to AAC, except for two cases in which the cause of death was unclear. This was confirmed by the fact that there was no significant difference in clinical efficiency between the non-death group and the death group (77.8% vs. 64.7%, P = 0.55) and most of the patients in both groups reported the relief of AAC symptoms after PC. Our multivariate analysis showed that a higher aCCI score was an independent predictor of 60-day mortality. This result supported the view of Huffman et al. that AAC was likely an epiphenomenon of overall critical illness, only occasionally being the main cause of death [1]. The clinical efficiency rate within 5 days after PC was 72.7%, which was lower than the 86.7% reported in the study of Noh. This may be related to the inclusion of mild AAC patients in the study by Noh. In addition, it may be related to the current indicators for evaluating the treatment effect of moderate to severe AAC were not suitable enough, because six patients had disorders of consciousness which caused the difficulty to judge the abdominal signs, thirteen patients developed AAC during hospitalization, some of whose uncontrolled comorbidities caused the improvement of temperature and WBC not obvious, or even increase after PC. Moreover, two patients in the death group left the hospital on postoperative day 1. Thus, we only obtained their indicators on the first day, which may have resulted in the clinical efficiency being underestimated. The resolution of AAC symptoms did not significantly lower mortality in our study. However, in a nationwide analysis of AC, after adjustment for baseline mortality risk, percutaneous cholecystostomy was associated with reduced mortality compared with no intervention [19].

The rate of PC-related complications in our study was only 4.5%, comparable to the rates reported in previous studies on the safety of PC (3–11%) [7]. All patients had moderate-to-severe AC in our study, which further confirmed that PC is a technically safe choice. The recurrence rate after treatment with PC alone was 22.2% (6/27), which was significantly higher than the rates of 8.6% reported in the study of Abbas [8] and 2.7% reported in the study of Noh [7] but lower than the rate of 40% (2/5) reported in the study of Polistina [20]. Compared with the study of Noh, the high recurrence rate observed in our study may be related to the exclusion of Grade I AAC patients. In our study, the rate of patients who died during indwelling drainage tubes was 38.6%, which made it impossible for us to obtain recurrence data from these patients. In addition, the limited sample size may be an important reason for the high recurrence rate noted in our study. Polistina et al. had a similar view. Our results cannot confirm that PC should be used as a definitive treatment for AAC patients who are unfit for surgery.

In our study, two patients (2/6, 33.3%) had recurrence during the indwelling drainage tube period. A few days before recurrence, bile drainage decreased significantly, but the color was normal. In addition, they had no jaundice. Their catheters were flushed with normal saline. The bile drainage gradually recovered after catheter flushing. This indicated that tube occlusion caused recurrence because the cystic duct and common bile duct were patent during recurrence. Catheters are susceptible to occlusion owing to viscous bile, blood clots, and stones [21]. In our study, the two recurrent patients had no gallstones, so it is more likely that viscous bile or blood clots caused the recurrence of AAC during the indwelling drainage tube period.

It is unclear how long the drainage tube should remain in place, and there are few related studies [5, 22]. We initially maintained the drainage tube for 2–3 weeks, but a few patients had abdominal pain caused by bile leakage, which may be related to the longer time needed for tract maturity in elderly patients. Therefore, we kept PC for at least 4 weeks during this study stage, and no bile leakage occurred in the patients whose tubes were removed in our institution. Our institution is a provincial class three grade A hospital, so nearly half of the patients come from the surrounding cities, and some of them removed their tubes at local hospitals. Therefore, it is difficult to obtain the drainage duration and PC clamping duration exactly, which prevented us from including the drainage and PC clamping duration in our analysis. To date, many studies on PC for acute cholecystitis have reported that the drainage duration of cholecystostomy tubes is related to the incidence of postprocedural morbidity [5, 23, 24].

The study has several limitations. The nature of this retrospectively designed single-center study limits its clinical value. Second, the recurrent population was relatively small in our study. However, it is a common phenomenon that the number of recurrent patients is relatively low. In a previous study, Noh et al. reported that only two of 88 AAC patients experienced recurrence of cholecystitis after catheter removal. In addition, moderate and severe AAC treated with PC in our institution exhibited a high short-term mortality rate, which further reduced the number of patients we could include in the recurrence analysis. Future large-scale studies should be performed to obtain a more representative population of recurrent patients. Furthermore, the lack of some data prevented some potential risk factors for AAC recurrence from being included in the study, such as tube clamping and tubogram before PC tube removal.

In conclusion, PC is effective for most patients with moderate and severe AAC during the acute phase. PC can be used as an important treatment for AAC. The presence of CAD or CHF at the time of PC placement was independently associated with the recurrence of AAC. In addition, preoperative aCCI scores were positively correlated with mortality after PC.

## Data Availability

The datasets used and/or analysed during the current study are available from the corresponding author on reasonable request.

## Abbreviations

AAC: Acute acalculous cholecystitis
ACC: Acute calculous cholecystitis
aCCI: Age-adjusted Charlson Comorbidity Index
ALT: Alanine aminotransferase
ASA-PS: American Society of Anesthesiologists physical status classification
CB: Conjugated bilirubin
CCI: Charlson Comorbidity Index
CHD: Coronary heart disease
CHF: Congestive heart failure
CI: Confidence intervals
CRF: Chronic renal failure
CT: Computed tomography
MRCP: Magnetic resonance cholangiopancreatography
OR: Odds ratio
PC: Percutaneous cholecystostomy
STB: Serum total bilirubin
UCB: Unconjugated bilirubin
US: Ultrasonography
WBC: White blood cells
